# Development of a multi-wear-site, deep learning-based physical activity intensity classification algorithm using raw acceleration data

**DOI:** 10.1371/journal.pone.0299295

**Published:** 2024-03-07

**Authors:** Johan Y. Y. Ng, Joni H. Zhang, Stanley S. Hui, Guanxian Jiang, Fung Yau, James Cheng, Amy S. Ha

**Affiliations:** 1 Department of Sports Science and Physical Education, The Chinese University of Hong Kong, Hong Kong, Hong Kong; 2 School of Public Health, The Chinese University of Hong Kong, Hong Kong, Hong Kong; 3 Department of Computer Science and Engineering, The Chinese University of Hong Kong, Hong Kong, Hong Kong; Ningbo University, CHINA

## Abstract

**Background:**

Accelerometers are widely adopted in research and consumer devices as a tool to measure physical activity. However, existing algorithms used to estimate activity intensity are wear-site-specific. Non-compliance to wear instructions may lead to misspecifications. In this study, we developed deep neural network models to classify device placement and activity intensity based on raw acceleration data. Performances of these models were evaluated by making comparisons to the ground truth and results derived from existing count-based algorithms.

**Methods:**

54 participants (26 adults 26.9±8.7 years; 28 children, 12.1±2.3 years) completed a series of activity tasks in a laboratory with accelerometers attached to each of their hip, wrist, and chest. Their metabolic rates at rest and during activity periods were measured using the portable COSMED K5; data were then converted to metabolic equivalents, and used as the ground truth for activity intensity. Deep neutral networks using the Long Short-Term Memory approach were trained and evaluated based on raw acceleration data collected from accelerometers. Models to classify wear-site and activity intensity, respectively, were evaluated.

**Results:**

The trained models correctly classified wear-sites and activity intensities over 90% of the time, which outperformed count-based algorithms (wear-site correctly specified: 83% to 85%; wear-site misspecified: 64% to 75%). When additional parameters of age, height and weight of participants were specified, the accuracy of some prediction models surpassed 95%.

**Conclusions:**

Results of the study suggest that accelerometer placement could be determined prospectively, and non-wear-site-specific algorithms had satisfactory accuracies. The performances, in terms of intensity classification, of these models also exceeded typical count-based algorithms. Without being restricted to one specific wear-site, research protocols for accelerometers wear could allow more autonomy to participants, which may in turn improve their acceptance and compliance to wear protocols, and in turn more accurate results.

## Introduction

Engagement in regular physical activity (PA) is associated with better health and well-being across individuals of all ages [1–3]. In particular, research has shown that even low volumes of PA, including those that could be accrued from daily activities, can lead to beneficial outcomes [3]. Monitoring PA accurately across a wide spectrum of activities is hence important for clinical, research, or even general self-monitoring purposes. With the rapid growth of technology and advanced computation methods [4], PA information can be captured using small, wearable digital devices with relative ease. As a result, these devices are widely used in the consumer market and research [5]. While they serve as a tool to monitor activity, research has shown that they could be used to increase PA and improve the health of users [6,7]. In research settings, accelerometers are frequently used as the PA measure of choice. Typically, these devices contain accelerometer modules that capture acceleration on multiple axes, and can be attached to different wear-sites, most commonly to participants’ hips or wrists. Traditionally, acceleration information measured by these devices are converted to device-specific units for further analyses [8,9]. More recently, researchers have shifted towards the analyses of raw acceleration data captured by the devices instead, as they are more information-rich, and could increase generalisability of data captured using devices of different models [10–12].

With much larger volumes of data being available, researchers have adopted machine learning approaches to derive algorithms that could improve accuracies to existing methods for PA detection [13–15]. When applied appropriately, machine learning could be used to identify patterns in large dataset that may not be apparent to humans. This is applicable to accelerometers as raw data are typically collected at multiple axes and at high frequencies (up to 100Hz). For example, machine learning methods have been applied for the detection of activity types and posture [16], gait patterns [17], and falls [18]. While these methods were shown to have respectable accuracy for intensity classification purposes, the majority of them were developed for data collected by devices attached to a specific wear-site (e.g., hip or wrist). Although studies have found that devices worn at different wear-sites could produce results that are strongly associated [19], applying an algorithm intended for a different wear-site could still endanger the validity of results. Previously, researchers could only rely on participants’ compliance to the protocol, with no means of counterchecking. As a solution to this problem, researchers [20] have developed wear-site detection algorithms to identify the placement of the devices. While such developed algorithms have respectable sensitivity and specificity (i.e., close to 90%), machine learning approaches may yield methods with even better accuracies. Further, if a non-wear-site-specific intensity classification algorithm could be developed, researchers may allow participants a higher level of autonomy in terms of where and how to wear the devices. Practically, having an additional choice of wear-site may reduce non-compliance [21], or even device loss due to misplacements.

Based on the above, in this study we developed and evaluated algorithms based on deep neural networks that are aimed to (a) determine the wear-site of accelerometers, and (b) detected PA intensities irrespective of wear-site. While algorithms specifically for adults and children were developed, we further explored whether a unified algorithm could be created for both groups. We further examined whether the inclusion of additional anthropometric information of participants could improve the accuracy of the estimates.

## Materials and methods

### Participants and procedures

All procedures of the study were reviewed and approved by the Joint CUHK-NTEC Clinical Research Ethics Committee (Ref. No. 2018.038). We recruited 60 healthy participants (30 adults and 30 children) to take part in the study between October 2018 and October 2019. Adult participants were recruited through university mass mail postings, and children were recruited through primary schools the authors had contact with. Adult participants provided initial consent using an online form before they were invited to the laboratory for data collection purposes. For participating children, information sheets were sent to their parents in advance, only those whose parents provided written consent were invited to the laboratory. Invited participants were asked to abstain from alcohol or coffee for eight hours prior to testing and to consume only water from three hours prior to testing. After participants arrived at the laboratory, research staff explained the procedures involved in the study, and asked participants to sign an informed consent form to confirm their participation. All invited participants agreed to the procedures and signed the consent form.

Participants height and weight were measured, and then they completed a semi-structured data collection protocol. Although all participants completed the protocol, there was data loss due to technical failures. As a result, data from six participants (four adults and two children) had to be removed from the final analyses. As such, the final analyses were conducted based on data collected from 26 adults (26.9±8.7 years; 11 female) and 28 children (12.1±2.3 years; 19 female). Demographic information of participants is presented in Table 1.

**Table 1:** Demographic background of participants and their metabolic exertion during different activity types.

	Adults	Children
Sex		
• Male	15	9
• Female	11	19
Age	26.9 ± 8.7 years	12.1 ± 2.3 years
BMI	21.8 ± 2.0 kg/m^2^	18.3 ± 2.5 kg/m^2^
Oxygen consumption (VO_2_) at different activity types (in metabolic equivalent)		
• Resting	4.09 ± 0.98 ml/kg/min (1.0 ± 0.0 METS)	5.71 ± 1.91 ml/kg/min (1.0 ± 0.0 METS)
• Living or light physical activity	6.86 ± 1.39 ml/kg/min (1.7 ± 0.9 METS)	9.81 ± 2.68 ml/kg/min (1.8 ± 1.0 METS)
• Moderate physical activity	22.91 ± 6.86 ml/kg/min (5.8 ± 1.9 METS)	25.58 ± 6.31 ml/kg/min (4.8 ± 1.5 METS)
• Vigorous physical activity	31.65 ± 8.15 ml/kg/min (8.2 ± 2.3 METS)	40.50 ± 8.52 ml/kg/min (7.7 ± 2.1 METS)

### Data collection protocol

**Fig 1:**
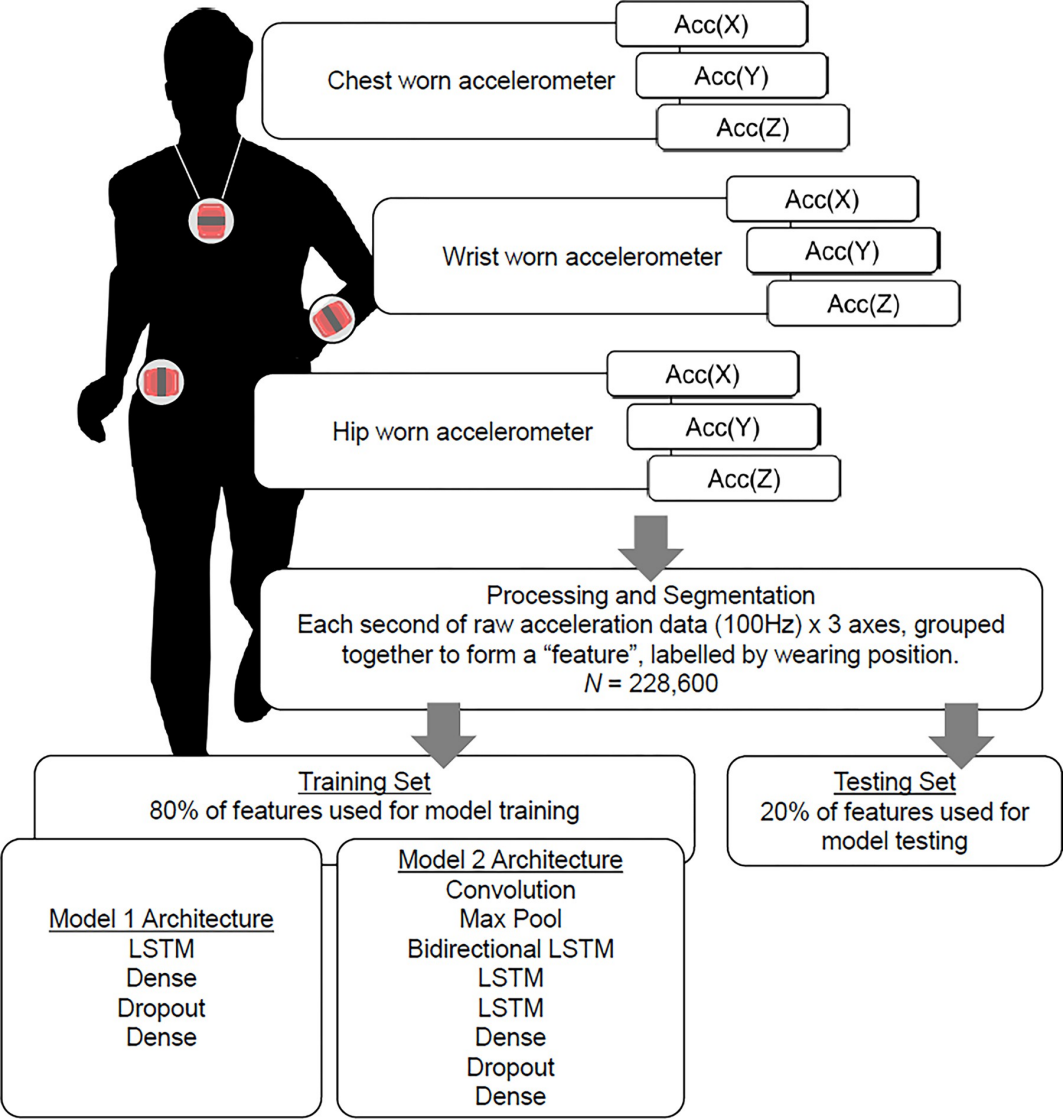
Schematic diagram of data collection and analyses procedures of study.

All activities took place in an exercise laboratory and were completed in a single visit. Research assistants first fitted the measurement devices on the participants. Participants were then asked to sit down with minimal movement for ten minutes so their resting heartrate and metabolic rate could be measured (resting rates were taken as the average between the sixth and ninth minute during the resting period). Each participant then performed 7 activities from a list of 17 choices. Activities were grouped into the following categories: (1) Sedentary or light daily living activities (e.g., sitting and writing, reading, playing a card-matching game, sewing, light effort sweeping, folding clothes, using a tablet computer while seated), (2) light physical activities (e.g., walking at 2 km per hour [kph], walking at 3.2 kph, stretching exercises), (3) moderate physical activities (e.g., walking at 4.0 kph, walking at 4.8 kph, walking at 4.8 kph at 3% inclination, following a video to do a moderate workout) and (4) vigorous physical activities (e.g., running at 6.4 kph, running at 7.2 kph, self-paced stair climbing). Cards labelled with each activity were shuffled and participants randomly drew three activities from category 1, three activities from category 2 or 3, and one activity from category 4. Each activity was performed for 5 minutes, or up to 8 minutes to ensure participants’ metabolic rates remained at a steady state for at least 3 minutes. Participants were given 3 minutes of rest time (or when subject’s heartrate returned to resting heartrate, whichever is sooner) between each activity. Their mean oxygen consumption and corresponding metabolic equivalents (METS) when performing these activities are presented in Table 1. A simple schematic diagram to demonstrate the data collection and analyses procedures is presented in Fig 1.

### Measures

#### Accelerometry

Tri-axial ActiGraph GT3X+ accelerometers (ActiGraph, Florida, U.S.A.) were attached to three wear-sites on the subjects: on the hip, wrist, and chest by suspending a device using a lanyard placed around the neck. The hip and wrist are common attachment sites used in extant research. The chest was also found to be a viable wear-site [22], and are applicable to some consumer devices. Therefore, it was included as a third wear-site in the study. Accelerometer data were recorded at a frequency of 100Hz. Raw acceleration data at the three axes were extracted and used for analyses. While some previous research have extracted additional features from these devices for analyses [15], in this study we only used raw acceleration data of the axes to ensure our results could be applied to all devices containing triaxial accelerometer modules. To ensure the extracted data were taken from a steady state of activity and metabolism, the first and final minute of data taken from each activity under the protocol were excluded from the analyses.

#### Heartrate and metabolic rate

Participants’ heartrate was measured using a Polar heart rate sensor (Polar Electro, Kempele, Finland) attached to the chest using an adjustable strap. The heart rate monitor was used in this study to monitor participants’ response to exercise (i.e., reaching steady state). The COSMED K5 (COSMED Srl, Rome, Italy) portable metabolic analyser used as the criterion to measure exercise intensity in this study. The COSMED K5 was used to measure oxygen consumption (VO₂ per kg) and carbon dioxide production during rest periods and activities within the study protocol. The average VO₂ per kg at steady state (i.e., no clear increasing or decreasing trends observed) during each activity was calculated. When a participant was unable to attain a steady state during an activity, the corresponding data (per activity) were excluded from the analyses. Metabolic equivalents for each activity were estimated by dividing the corresponding oxygen consumption with that at resting stage. Activities per participant were then classified to sedentary, light, moderate, or vigorous using the cutoff values of METS < 1.5, 1.5 ≤ METS < 3.0, 3.0 ≤ METS < 6.0, and METS ≥ 6.0, respectively. These activity intensities were used as the ground truth in our analyses.

### Data analyses

To demonstrate the accuracy of the results derived from deep neural network models compared to existing count-based methods of intensity classification, activity intensities were first calculated using standard hip-worn protocols for adults [9] and children [8], respectively. To further demonstrate how misspecified wear-sites might negatively affect the accuracy of the estimates, we also applied the same protocols on data collected from wrist- and chest-worn devices. These preliminary results would serve as reference values for comparison when evaluating the performances of more advanced machine learning models.

The Long Short-Term Memory (LSTM) machine learning approach was used in this study. Analyses were conducted within the TensorFlow (Google) environment. LSTM is a class of recurrent neural networks in the field of deep learning, and it has shown to be superior for learning and forecasting time series datasets (e.g., continuous time stamped accelerometer data)[23]. LSTM methods have also shown high accuracies for developing activity classification and recognition algorithms in accelerometry [23,24]. Two models were tested for each series of analyses in this study: a) a simple LSTM model composed of one fully connected LSTM layer, one dense layer with ReLu (Rectified Linear Unit) activation and dropout, and one dense output layer with Softmax activation, and b) a stacked LSTM model (“Bi-LSTM”) composed of one convolution layer, one pooling layer, one bidirectional LSTM layer, two LSTM layer, one dense layer with ReLu activation and dropout, and one dense output layer with Softmax activation. Additional bi-directional models were applied to enhance data processing by capturing dependencies of data from both the past and future. By processing sequential data in both directions, the performances of neural networks may improve, and hence were applied in this study.

Prior to training and testing the models, the data was segmented using a sliding window with a size of 100 continuous samples (i.e., each window contained data from 1 second). Subsequently, a total of 228,600 sample features, labelled by wear-site, participant height and weight, and activity intensity were used for analysing the LSTM-based models. To evaluate the model, 90% sample features were randomly used to train the models (i.e., training set) and the remaining 10% was used to evaluate the performance of the models (i.e., validation set). Two sets of models were evaluated, respectively, for algorithms to classify wear-site and activity intensity. Both models were evaluated for two scenarios: (i) classification between two wear-sites (hip or wrist), and (ii) classification between three wear-sites (hip, wrist or chest). A training epoch of 20 was used for model training. The performances of the models were evaluated based on their percentage of correct classifications compared to the ground truth.

## Results

### Preliminary analyses

We first compared the PA intensities derived from existing hip-worn, count-based cut points for adults [9] and children [8], respectively, with the ground truth. The overall accuracy was 84%, which was similar to existing machine-learning based algorithms [15]. However, the corresponding specificities for some combinations of populations and activity intensities (e.g., children’s moderate PA, 35%; adults’ light PA, 51%) were low (Table 2). Further, if the device was positioned at a different wear-site, and researchers applied the same algorithm without the knowledge, the accuracy would further decrease (wrist-worn: 65%; chest-worn: 75%). These results suggest that misspecification of accelerometer wear-site could lead to low accuracies in PA intensity detection.

**Table 2:** Accuracy of activity intensity classification using count-based algorithms in this study.

Group	Ground truth intensity	Count-based algorithms								
		Hip-worn			Wrist-worn *			Chest-worn *		
		‐	=	+	‐	=	+	‐	=	+
Adult	Sedentary	–	99%	1%	–	76%	24%	–	96%	4%
	Light	36%	51%	13%	1%	44%	55%	14%	81%	5%
	Moderate	10%	78%	12%	23%	51%	27%	67%	29%	4%
	Vigorous	26%	74%	–	26%	74%	–	56%	44%	–
Children	Sedentary	–	99%	1%	–	74%	26%	–	89%	11%
	Light	25%	60%	16%	2%	31%	67%	9%	82%	9%
	Moderate	40%	35%	25%	20%	33%	47%	68%	25%	8%
	Vigorous	1%	99%	–	14%	86%	–	29%	71%	–
Adult	Combined	11%	85%	4%	8%	67%	26%	21%	75%	3%
Children	Combined	10%	83%	7%	6%	64%	31%	17%	74%	9%
Combined	Combined	10%	84%	6%	7%	65%	28%	19%	75%	6%

Notes.

* Hip-worn algorithms were applied to simulate misspecification of wear-site. ‐ Underestimated activity intensity; = Correctly estimated activity intensity; + Overestimated activity intensity.

### Main analyses–wear-site classification

The models trained using deep neural network showed respectable levels of accuracy when predicting between the hip and wrist wear-sites (LSTM model: 93.0%, Bi-LSTM: 93.7%; see Table 3 for detailed results). There were slight differences between the accuracies of trained models for children (LSTM: 93.2%; Bi-LSTM: 93.6%) and adults (LSTM: 95.3%; Bi-LSTM: 96.5%). When the models were trained to classify between three wear-sites (hip, wrist, and chest), the accuracies obtained were lower numerically, but were generally above 90% (for the combined adult and children sample; LSTM: 91.1%; Bi-LSTM: 92.8%).

**Table 3:** Performances of deep neural networks developed in the study.

Purpose	Group	Hip and wrist wear-sites only		Hip, wrist, and chest wear-sites	
		LSTM	Bi-LSTM	LSTM	Bi-LSTM
Wear-site detection	Adult	95.3%	96.5%	88.3%	90.8%
	Children	92.6%	93.6%	89.5%	92.1%
	Combined	93.0%	93.7%	91.1%	92.8%
Intensity classification	Adult	92.0%	93.0%	86.5%	88.4%
	Children	89.8%	90.3%	86.3%	87.6%
	Combined	90.8%	91.3%	86.5%	86.2%
Intensity classification with age, height, and weight as additional parameters	Adult	96.0%	97.1%	93.3%	94.5%
	Children	95.7%	95.4%	93.8%	92.7%
	Combined	96.2%	96.3%	93.9%	92.8%

### Main analyses–activity intensity classification

Models were also trained to classify activity to intensities of sedentary, light, moderate, or vigorous. The predictive accuracies of the trained models, when the possible wear-site was limited to two, was around 90% (for the combined adult and children sample; LSTM: 90.8%; Bi-LSTM: 91.3%). Similar to the wear-site specification model, there were slight differences between the accuracies for the models for adult- or children-specific data (please refer to Table 3 for details). We further examined whether the inclusion of labels for participants height, weight, and age would further improve the accuracies of the classifications. Results showed that with these inclusions, the accuracy of models increased beyond 95% (for the combined adult and children sample; LSTM: 96.2%; Bi-LSTM: 96.3%).

When using data collected from devices placed at all three wear-sites, the trained models correctly predicted the intensities of the activity below 90%, when no additional anthropometric information was included (for the combined adult and children sample; LSTM: 86.5%; Bi-LSTM: 86.2%). When labels for participants height, weight, and age were included, the accuracies of the predictions increased to above 90% (for the combined adult and children sample; LSTM: 93.9%; Bi-LSTM: 92.8%).

## Discussion

In the current study, we applied deep neural network to develop classification algorithms for the detection of accelerometer wear-site and PA intensity. Our results showed that one-second samples of raw accelerations contain sufficient information for classification purposes, with the algorithm correctly determining the wear-site on approximately 90% of the time. While researchers [20] have shown that a similar level of specificity was achievable through non-machine learning approaches, the analyses of data from longer windows was required. Based on existing accelerometer deployment protocols, participants are usually recommended to adhere to a single protocol for accelerometer wearing, and therefore longer windows are practical. In fact, one may argue that second-by-second classifications of wear-site is unpractical given changes in wear-site, if any, do not happen rapidly or frequently. Nonetheless, the current study is a strong proof of concept, and new algorithms could be developed by stacking second-by-second classifications to longer windows that would receive the same wear-site label.

As demonstrated in our preliminary analyses, our intensity classification models outperformed results derived from count-based approaches (91% versus 84%). Nonetheless, data collection for this study was conducted in a controlled, laboratory environment, whereby the researchers can determine the wear-site with certainty. If, instead, devices were administered in the field and the participants placed the device at a different wear-site, and the researcher processed the data unknowingly, the difference in accuracy could be even more substantial (91% versus 65%). Yet researchers have shown that when appropriate algorithms were applied, hip- and wrist-worn devices can provide very similar estimates [21]. Therefore, the ability to correctly detect device wear-site is, on its own, of great importance to ensure the validity of accelerometer-based PA data. Nonetheless, in this study, we have further developed models for intensity classification based on data collected from devices, irrespective of their wear-sites. In a similar study, Montoye and colleagues [14] simultaneously collected data from multiple devices and used artificial neural networks to develop intensity classification algorithms for the respective wear-sites. However, to the best of our knowledge, our study was the first successful attempt to create a non-wear-site-specific method for intensity detection, which has great potential for wide adoption in PA and health research.

Another advancement of the current study was the inclusion of the chest wear-site. Although chest-worn protocols for accelerometers [22] are uncommon in research, necklace-attached activity trackers are common in consumer devices. In fact, during the COVID-19 pandemic, some participants (or parents of children) were sceptical to the idea of attaching a device in direct contact with their bodies. Providing a chest-worn option may potentially increase acceptability to data collection protocols. Adherence may also improve given its relative ease of use. We do not, nevertheless, imply that the three included wear-sites are the only viable placement sites. By contrast, the results of our study suggest that advanced modelling approaches could allow for the development of unifying algorithms for any wear-sites. For example, the thigh [25], ankle [26], and ears [27] are all possible placement sites that could be incorporated in research in the future. Results from this study could have major implications to research data collection protocols. For example, instead of requiring participants to adhere to a single wear-site, or even requesting them to switch between during non-sleeping and sleeping periods [28], researchers could allow participants to choose, and switch, between several wear locations based on their preferences, expected activities, or choice of attire. In fact, these are common requests made by participants in real-life settings. Providing such autonomy to participants could potentially improve their adherence, thereby reducing non-adherence and potential reduce device loss due to misplacement of devices.

While a universal (in terms of wear-site and participant age group) activity intensity classification algorithm based on accelerometer-data alone may be coveted, results of our study suggested that the predictive power of the models can be improved when additional features, such as participants age, height, and weight are also incorporated into the models. Specifically, with these additional parameters included, the accuracies of the predictions could reach over 95%. Based on these findings, it might be appropriate to postulate that other characteristics of participants, such as their anthropometric measures, are necessary inputs for the development of a unified activity intensity detection algorithm for all age groups. While we did include both children and adult participants in our study, future studies should incorporate participants from more age groups (e.g., adolescents, older adults) and examine what parameters, such as age or anthropometric measures, might be critical to the predictive powers of such models. Nonetheless, researchers should continue to examine the performances of algorithms by separating and combining, respectively, data from participants of different age groups, to investigate the feasibility of developing non-age group-specific models for activity intensity classification. Also, in our study, a sampling frequency of 100 Hz was used for accelerometer data retrieval in our study to maximize the information that could be captured and used. While researchers agreed to maximizing the volume of data retrieval in such studies [15], more processing power will be required for data analysis. Further work is still required to explore how to optimize the balance between richness and efficiency in processing data.

### Limitation

A limitation of the study is that all activities were conducted under a controlled environment within a laboratory. Findings from laboratory-based studies were not always reproducible in free-living settings [13]. The limited choice of activities may also be insufficiently diverse to represent all possible forms of activities at all intensities. Hence additional research is needed to evaluate similar methods in non-laboratory settings. Furthermore, we were unable to identify potential factors that might contribute to the accuracies of prediction models. Further research is required to examine if other participant characteristics, including but not limited to sex, age, fitness levels, or skill level with respect to the activity tasks might affect the predictive accuracies of the models.

## Conclusions

In this study, we provided initial evidence that non-wear-site-specific activity classification algorithms for accelerometer data can be derived using deep neural networks. Furthermore, by applying similar data analytical approaches, we were able to correctly classify activity intensity above 85% of the time, without prior knowledge of the device wear-site. While additional work may be required to explore its application in real-life settings and adoption in research studies, our results may serve as important reference for future research in this direction. Findings from this study could have important implications to protocols for the collection of physical activity data, which may improve participant adherence and in turn, the quality and quantity of data available for research.
